# Supplementary material for: WordCommentsAnalyzer: A windows software tool for qualitative research
Source: F1000Res. 2018 Sep 4;7:536. Originally published 2018 May 3. [Version 2] doi: 10.12688/f1000research.14819.2 (PMC6137411; doi:10.12688/f1000research.14819.2)
Supplement: Supplementary file 3 [file f1000research-7-17614-s0002.tgz › b2a79496-4ada-47c4-9682-a99ce867836d.zip!/Eight Iranian Health Journals/Yafteh _29.docx]

© Yafteh  . This work is licensed under CC BY 4.0 (https://creativecommons.org/licenses/by/4.0/).

Volume & Issue: دوره 13 - شماره 3

Title: بررسی تاثیر اجتماع درمان مدار روزانه در ویژگی‌های شخصیتی بیماران معتاد مراجعه کننده به مرکزTC شهر خرم‌آباد

http://yafte.lums.ac.ir/article-1-555-fa.html

مقدمه: سوءمصرف مواد و اعتیاد امروزه یک معضل اجتماعی و درمانی است که تبعات آن در جامعه بسیار سنگین است . برنامه‌های مختلفی جهت درمان و بازتوانی بیماران وابسته به مواد افیونی وجود دارد. تحقیقات انجام شده نشان داده اند که روش اجتماع درمان‌مدار شیوه مناسبی برای بازگرداندن افراد معتاد به جامعه می‌باشد . در مطالعه حاضر بر آن شدیم تا تاثیر برنامه‌های درمانی اجتماع درمان‌مدار (TC) را در ویژگی‌های شخصیتی افراد وابسته به مواد مورد ارزیابی قرار دهیم . مواد و روش‌ها: مطالعه حاضر یک مطالعه مداخله‌ای است . جامعه مورد مطالعه شامل کلیه معتادان مرد است که دوره سم زدائی در آنها به پایان رسیده است. نمونه ها به روش تصادفی ساده ازبین افراد مراجعه کننده به مرکز TC انتخاب و در مطالعه مورد بررسی قرار گرفتند. در ابتدای ورود به مطالعه پرسشنامه SCL 90 توسط روانشناس حهت کلیه واحدهای مورد پژوهش تکمیل گردید . نمونه ها بر اساس فرمول حجم نمونه به تعداد 60 انتخاب شدند .بیماران به مدت سه ماه، هر روز به غیر از روزهای جمعه دردو شیفت صبح و عصر تحت برنامه های آموزشی TC قرار گرفتند . در پایان دوره سه ماهه مجددا آزمون SCL 90 جهت کلیه نمونه ها اجرا و اطلاعات پس از جمع آوری مورد تجزیه و تحلیل قرار گرفت . بحث و نتیجه‌گیری: همچنان که در یافته‌های تحقیق ذکر شد انجام مداخلات TC بطور معنی‌داری در نمرات اختلالات شخصیت موثر بود . پیشنهاد می‌گردد متولیان، دست اندرکاران و سیاست گزاران مبارزه با اعتیاد کشور با تقویت مراکز اجتماع درمان‌مدار در دست یافتن به زندگی بدون مواد و درمان اختلالات شخصیت در معتادان اهتمام ورزند . یافته‌ها: نتایج بررسی بیماران قبل از ورود به مطالعه نشان داد 7/16 % سالم ، 40 % اختلال شخصیت در حد مرزی ، 30 % بیمار و 4/13 % به شدت بیمار و بعد از انجام مداخلات TC 25 % سالم ، 7/66 % اختلال شخصیت در حد مرزی و 3/8 % بیمار بودند . هیچ یک از بیماران بعد از انجام برنامه های TC به شدت بیمار نبودند .میانگین نمرات اختلال شخصیت قبل از مداخلات TC 92/0 ± 40/2 و بعد از انجام مداخلات TC 56/0 ± 83/1 بود. آزمون رتبه ای ویلکاکسون تفاوت معنی داری بین شدت اختلالات شخصیتی قبل و بعد از انجام مداخلات TC نشان داد(001/0 >p).

Title: بررسی جهش‌های ژن N-RAS در اطفال مبتلا به لوسمی لنفوبلاستیک حاد(ALL)

http://yafte.lums.ac.ir/article-1-556-fa.html

مقدمه: لوسمی حاد شایعترین بدخیمی کودکان است. یکی از مهم‌ترین عوامل ایجاد کننده لوسمی لنفوبلاستیک حاد(Acute Lymphoblastic Leukemia) جهش های مربوط به پروتوانکوژنها و تبدیل آنها به انکوژن ها می باشد. از جمله این پروتوانکوژنها، پروتوانکوژن N-RASاست. از آنجایی که آماری از فراوانی جهش این ژن در کودکان ALL ایرانی وجود نداشت بر آن شدیم میزان فراوانی این جهش را در تعدادی از این بیماران بررسی و ارتباط این جهش ها را با سن، جنس و نوع ALL بررسی نماییم. بحث و نتیجه‌گیری: نتایج بدست آمده در این تحقیق از نظر فراوانی جهش با آمار ارائه شده توسط سایر محققان تفاوت چندانی نداشت. یافته‌ها: جهش‌های N-RAS در 7/11% (60/7) بیماران شناسایی شد، بیشتر آنها مذکر و کمتر از5 سال بودند. فراوانی کلی جهش ها در کدونهای 12، 13 و 61 به ترتیب 3/8%، 3/3% و7/1% به دست آمد و بیشتر بیماران دارای جهش (4/71%) c-ALL بودند مواد و روش‌ها: در این مطالعه توصیفی 60 نفر کودک بیمار با تشخیص ALL ) 32نفر common-ALL، 20نفر preBALL ،6 نفر T-ALL و2 نفر BALL ( با طیف سنی 11 ماهه تا 14 سال،شامل 40 نفر مذکر 20 نفر مونث از نظر جهش های ژن N-RAS در کدون‌های 12، 13 و 61 با روش PCR-RFLP مورد بررسی قرار گرفتند.

Title: بررسی هیدروکسی پرولین درسوسیس تولیدی کارخانجات خرم‌آباد

http://yafte.lums.ac.ir/article-1-557-fa.html

مقدمه: محصولات گوشتی حرارت دیده از جمله سوسیس یکی از پر طرفدارترین محصولات گوشتی در ایران می باشد. این امر اهمیت کنترل و شناسایی تقلبات را در آنها به منظور تامین امنیت غذایی مشخص می کند.هدف از این مطالعه تعیین میزان هیدروکسی پرولین در سوسیس تولیدی کارخانجات شهر خرم آباد در تابستان سال 1388 بود. بحث و نتیجه‌گیری: بعنوان نتیجه گیری کلی می توان گفت مقدار هیدروکسی پرولین، کلاژن و ازت کلاژنی سوسیس تولید شده در کارخانه گلبهار خرم آباد در تابستان 1388 کمتر از میزان استاندار است .بنابراین احتمال تقلب در آن کم به نظر می رسد. یافته‌ها: میانگین هیدروکسی پرولین در100 گرم نمونه 017/0 و انحراف معیار آنها 009/0 بود. این مقدار بسیار کمتر از میزان استاندارد است. مقدار کلاژن و ازت کلاژنی به ترتیب 07/0±13/0 و 01/0± 02/0 گرم در 100 گرم نمونه بود. مواد و روش‌ها: این مطالعه مقطعی بر روی تعداد 30 نمونه سوسیس تولید شده در کارخانه گلبهار شهرستان خرم آبادانجام شد. نمونه ها از یک خط تولید و بر اساس اصول نمونه گیری انتخاب و در شرایط مناسب به آزمایشگاه منتقل شد.سپس بر اساس SOP روش آزمون که در آزمایشگاه موجود بود و با روش اسپکتروفتومتری آزمایش تعیین میزان هیدروکسی پرولین انجام شد. مقدار کلاژن و ازت کلاژنی نیز از طریق فرمول محاسبه شد.روش گرد آوری اطلاعات مشاهده ای بود. داده ها در نرم افزار SPSS وارد و با استفاده از آمار توصیفی و همچنین آزمون t یکطرفه ( مقایسه نتایج با عدد استاندارد) تجزیه و تحلیل شد.

Title: تأثیر روش آرام سازی پیشرونده عضلانی بر اضطراب بیماران مبتلا به سکته قلبی

http://yafte.lums.ac.ir/article-1-559-fa.html

مقدمه: بیماری‌های قلب وعروق از شایع‌ترین بیماریهای موجود در جوامع بشری محسوب می‌شوند.با توجه به وجود استرس‌های فراوانی که پس از سکته قلبی به وجود می‌آید و نیز تاثیری که اضطراب می‌تواند بر پیامدهای درمانی بیماران مبتلا به سکته قلبی داشته باشد، مطالعه حاضر با هدف بررسی تاثیر به کارگیری روش آرام‌سازی پیشرونده عضلانی بر اضطراب بیماران مبتلا به سکته قلبی انجام شد. بحث و نتیجه‌گیری: آرام سازی پیشرونده عضلانی می تواند باعث کاهش میزان اضطراب، فشارخون سیستولیک و دیاستولیک در بیماران مبتلا به سکته قلبی بستری در CCU گردد. لذا این روش می تواند به عنوان یک درمان غیر دارویی مکمل، ساده و کم هزینه در درمان این بیماران نقش موثری ایفاء نماید. یافته‌ها: در گروه مداخله میزان اضطراب، فشار خون سیستولیک و دیاستولیک در مقایسه با گروه کنترل کاهش معنی داری داشت (001/0P<). مواد و روش‌ها: این مطالعه به روش کارآزمایی بالینی بر روی 60 بیمار صورت گرفت که طی دو هفته پیش از شروع پژوهش مبتلا به انفارکتوس حاد میوکارد شده بودند. پس از انتخاب نمونه های واجد شرایط، بیماران به دو گروه مداخله و کنترل تقسیم شدند که از نظر سن، جنسیت و میزان اضطراب همسان سازی شده بودند. در هر گروه ابتدا در روز اول پرسشنامه اضطراب کتل توسط بیماران تکمیل و علائم حیاتی ثبت گردید. سپس در گروه مداخله، طی سه روز متوالی و مجموعاً در6 جلسه، تکنیک آرام سازی عضلانی پیشرونده انجام شد و مجدداً پس از سه روز متغیرها در هر دو گروه سنجیده شد. اطلاعات بدست آمده پس از جمع آوری مورد تجزیه و تحلیل قرار گرفت. برای تجزیه و تحلیل داده ها از آمار توصیفی و آزمون های tمستقل و t زوجی استفاده شد.

Title: بررسی اثرات مصرف آبغوره بر عوامل خطر پروفایل لیپید و پیشرفت آترواسکلروز در خرگوش‌های هایپرکلسترولمیک

http://yafte.lums.ac.ir/article-1-560-fa.html

مقدمه: امروزه نقش آنتی اکسیدانها در جلوگیری و بهبود بیماری آترواسکلروز به اثبات رسیده است. در این مطالعه اثرات مصرف آبغوره بر عوامل خطر پروفایل لیپید و پیشرفت آترواسکلروز در خرگوشهای‌ها یپرکلسترولمیک بررسی شده است. بحث و نتیجه‌گیری: نتایج این مطالعه پیشنهاد کننده تأثیر حمایتی مصرف آبغوره (بعنوان یک آنتی اکسیدان) بر روی برخی از عوامل خطر آترواسکلروز می‌باشد. یافته‌ها: مصرف روزانه ml 5 و 10 آبغوره (یک بار) افزایش معنی داری را در سطح ظرفیت آنتی اکسیدانی و HDL-C نسبت به گروه رژیم پرکلسترول نشان داد .هر دو دوز آبغوره موجب کاهش معنی‌دار در میزان ترانس آمینازهای کبدی ((SGPT,SGOT ،گلوکز و ApoB شد. مصرف ml 10 آبغوره موجب کاهش معنی‌دار در سطح TG نسبت به رژیم پرکلسترول شد. هیچ اختلاف معنی‌داری در سطح کلسترول تام و ApoA بین گروه‌های آبغوره و گروه رژیم پرکلسترول دیده نشد. نتایج بافت شناسی نشان داد که مصرف آبغوره به میزان زیادی موجب کاهش شدت ضایعه در دیواره آئورت نسبت به گروه پرکلسترول شد. مواد و روش‌ها: 32 خرگوش نر نیوزیلندی بطور تصادفی به 4 گروه دریافت کننده تقسیم شدند؛ 1- معمولی 2- پرکلسترول (1% کلسترول) 3- پرکلسترول (1% کلسترول) و ml 5 آبغوره 4- پرکلسترول (1% کلسترول) و ml 10 آبغوره.فاکتورهای گلوکز، کلسترول تام (TC )، تری گلیسرید (TG )، آپو لیپو پروتئین های A ,B (ApoA, ApoB )، ترانس آمینازهای کبدی (SGPT ,SGOT )، لیپوپروتئین با دانسیته بالا (HDL-C ) و ظرفیت آنتی اکسیدانی قبل از آزمایش و دو ماه پس از تیمار اندازه گیری شد. در انتهای مطالعه، تشکیل رگه چربی در آئورت در همه گروهها تعیین و بررسی شد.

Title: بررسی تاثیر سولفات روی خوراکی بر رشد شیرخواران نارس

http://yafte.lums.ac.ir/article-1-561-fa.html

مقدمه: روی یک ریز مغذی است که نقش بسزایی در رشد کودکان دارد و عمده آن در سه ماهه سوم حاملگی وارد بدن نوزاد می شود لذا نوزادان پره ترم به میزان کافی آن را دریافت نکرده و ذخایر کمتری برای روی دارند. هدف از این مطالعه تعیین تاثیرسولفات روی خوراکی بر رشد شیرخواران نارس بود. بحث و نتیجه‌گیری: تجویز سولفات روی به شیر خواران پره ترم به میزان 3 میلی گرم روزانه تا پایان 6 ماهگی اثرات چشمگیری در افزایش وزن ، قد و دور سر دارد و می‌توان برای جبران کمبودهای رشدی در شیرخواران نارس از روی استفاده نمود. یافته‌ها: میانگین سن موقع تولد در گروه اول(2/2 ±9/32)هفته و در گروه دوم (1/2±7/32) هفته بود. میانگین وزن در گروه دریافت‌کننده روی در پایان 6 ماهگی(3/810±3/6861)گرم و در گروه کنترل(7/534±9/5118)گرم بود (001/0 >p) . از نظرافزایش میزان قد و دور سر تا پایان 2 ماهگی بین دو گروه تفاوت قابل توجهی وجود نداشت، ولی از ماه سوم گروهی که روی دریافت کرده بودند در مقایسه با گروه کنترل تفاوت چشمگیری داشتند. میانگین قد در پایان 6 ماهگی درگروه اول(8/3±8/59 (سانتیمتر و در گروه دوم(5/3±0/57 (سانتیمتر ، میانگین دور سر نیز در گروه اول(0/2±4/41)سانتیمترو گروه دوم (5/2±5/39) سانتیمتر بود (001/0 >p) . هیچگونه عارضه جدی در طول تجویزمشاهده نشد. مواد و روش‌ها: این مطالعه بر روی 106 نوزاد پره ترم با سن حاملگی 36 -28هفته و وزن 2500-1000 گرم که در بیمارستانهای امام رضا (ع) و معتضدی کرمانشاه متولد شده بودند انجام شد.نوزادان به صورت کاملا تصادفی به دو گروه 53 نفره تقسیم شدند.به گروه اول روزانه 3 میلی گرم شربت سولفات روی به مدت 6 ماه داده شد ولی گروه دوم روی دریافت نکردند. هر دو گروه تا پایان 6 ماهگی به صورت ماهیانه مورد معاینه و سنجش وزن، قد و دور سر قرار گرفتند.

Title: بررسی میزان آگاهی فراهم‌کنندگان مراقبت بهداشتی از جنبه‌های قانونی مدارک پزشکی در بیمارستان‌های آموزشی وابسته به دانشگاه علوم پزشکی لرستان

http://yafte.lums.ac.ir/article-1-562-fa.html

مقدمه: آگاهی فراهم کنندگان مراقبت بهداشتی از قوانین حاکم در بهداشت و درمان در ارتباط با پرونده های پزشکی و اطلاعات بهداشتی بیماران، شرایط تامین حقوق بیمار را فراهم می کند؛ از این‌رو این مطالعه به منظور تعیین میزان آگاهی ارائه دهندگان مراقبت از جنبه‌های قانونی مدارک پزشکی در بیمارستان‌های آموزشی شهر خرم‌آباد در سال 1389 صورت پذیرفت. مواد و روش‌ها: این تحقیق به روش توصیفی – مقطعی روی 210 نفر از فراهم کنندگان مراقبت بهداشتی ( پزشکان، پرستاران و پرسنل بخش مدارک پزشکی ) که بطور تصادفی انتخاب شده بودند انجام گرفت. داده ها با پرسشنامه ای که بر اساس موارد قانونی مرتبط با اطلاعات بهداشتی طبق کتب مرجع و نظر کارشناسان مربوطه تهیه شده بود گردآوری و با استفاده از نرم افزارSPSS و با شاخص‌های درصد فراوانی و میانگین و انحراف معیار امتیازات تحلیل شد. بحث و نتیجه‌گیری: میزان آگاهی از جنبه‌های قانونی مدارک پزشکی در حد متوسط است که این امر لزوم آموزش جنبه‌های قانونی مدارک پزشکی به‌خصوص صدورگواهی پزشکی را در رده‌های شغلی مختلف به‌خصوص پرستاری می‌رساند. یافته‌ها: میزان آگاهی فراهم‌کنندگان مراقبت از جنبه‌های قانونی مربوط به محرمانگی با امتیاز 13/1 ± 79/3 از 9 ؛جنبه‌های قانونی اخذ رضایت‌نامه با امتیاز 62 /1 ± 47/4 از 10 و جنبه‌های قانونی مربوط به صدور گواهی با امتیاز 52/1± 40/3 از 11 در گروه آگاهی متوسط بود. و به‌طورکلی میزان آگاهی فراهم‌کنندگان مراقبت با امتیاز 53/4 ± 72/11 از 30 در سطح متوسط بود. پزشکان بیشترین آگاهی و پرستاران کمترین آگاهی را دارا بودند.

Title: ارتباط بین چاقی و سطح چربیهای سرم در مردان بالغ 34-18 ساله منطقه 6 تهران: مقایسه نمایه توده بدن، دور کمر و نسبت دور کمر به دور باسن

http://yafte.lums.ac.ir/article-1-563-fa.html

مقدمه: بیماریهای قلبی و عروقی از مهمترین عوامل مرگ و میر و از عمده ترین مشکلات بهداشتی در کشورهای پیشرفته و در حال پیشرفت از جمله کشور ما ایران می باشند. این مطالعه با هدف تعیین ارتباط بین چاقی و سطح چربی‌های سرم در مردان بالغ 34-18 ساله منطقه 6 تهران و مقایسه شاخص‌های تعیین کننده چاقی در جمعیت مورد مطالعه انجام شد. بحث و نتیجه‌گیری: BMI احتمالا می‌تواند به عنوان یک عامل پیش بینی کننده بیماریهای قلبی و عروقی در جامعه ایرانی به کار رود. برای رسیدن به نتیجه قطعی تر در مورد WC و WHR باید مطاله‌ای با حجم نمونه بالاتر انجام گیرد. یافته‌ها: در جامعه مورد بررسی 8/24% افراد مبتلا به هیپرکلسترومی، 8/29% مبتلا به هیپرتری‌گلیسریدمی، 8/63 % دارای LDL بالاتر از mg/dL 130و 7/78% دارای HDL کمتر از mg/dL0 4 بودند. BMI افراد مورد بررسی با مقادیر کلسترول تام، تری گلیسرید و LDL سرم همبستگی مثبت و معنی داری داشت. مواد و روش‌ها: مطالعه حاضر بر روی 141 مرد 34-18 ساله، با استفاده از لیست مدارس منطقه 6 تهران به عنوان سرخوشه و انتخاب نمونه ها از ساکنین اطراف این مدارس انجام گردید. سپس با مراجعه به منزل آنها ارزیابی های لازم (پرسشنامه ای، تن سنجی) انجام و تاریخ جمع آوری نمونه های خون تعیین گردید. نمونه های خون بین ساعت 8 تا 30/8 صبح با گرفتن 5 سی سی خون ( پس از 12 ساعت ناشتا بودن) انجام گرفت. پردازش آماری داده ها با نرم افزار SPSS و آزمون مجذورکای و ضریب همبستگی پیرسون انجام شد.

Title: عصاره هیدروالکلی برگ زیتون در مهار نفروتوکسیسیتی ناشی از جنتامایسین

http://yafte.lums.ac.ir/article-1-564-fa.html

مقدمه: نفروتوکسیستی حاصل از جنتامایسین مصرف این دارو را با محدودیت روبروکرده است . مطالعات متعددی نشان داده اند که رادیکال های آزاد در نفروتوکسیسیتی ناشی از جنتامایسین دخالت دارند. در برگ درخت زیتون بیو فنل هایی مانند اولئروپین با خواص آنتی اکسیدانی وجود دارد. برای اولین بار در تحقیق حاضر اثر عصاره هیدروالکلی برگ زیتون برنفروتوکسیسیتی ناشی از مصرف جنتامایسین بررسی گردید. مواد و روش‌ها: 35 موش صحرایی نر ن‍ژاد اسپراگ داولی بطور تصادفی به 5 گروه مساوی تقسیم شدند. به اولین گروه(گروه کنترل) تنها نرمال سالین تزریق شد. در چهارگروه نفروتوکسیسیتی با تزریق جنتامایسین(mg/kg 100) به مدت 12 روز ایجاد شد. سه گروه از موش ها همزمان با جنتامایسین تحت درمان با عصاره هیدروالکلی برگ درخت زیتون(دوزهای mg/kg 100 و50، 25) قرار گرفتند. در پایان روز دوازدهم رت ها کشته شده، غلظت سرمی مالون دی آلدئید(MDA)،اوره و کراتی‌نین اندازه گیری و از کلیه ها برای مطالعات هیستوپاتولوژیکی مقاطع بافتی تهیه و به روش پاس رنگ آمیزی گردید . بحث و نتیجه‌گیری: عصاره هیدروالکلی برگ درخت زیتون قادر به حفاظت در برابر نفروتوکسیسیتی ناشی از مصرف جنتامایسین است. احتمالاً این اثر حفاظتی به خواص آنتی اکسیدانی این عصاره وابسته است. یافته‌ها: عصاره هیدروالکلی برگ زیتون در هر سه دوز مصرفی به طور معنی‌داری افزایش نکروز توبولی و کاهش دانسیته حجمی لوله های خمیده نزدیک ناشی از مصرف جنتامایسین را مهار کرد (05/0 >P). تنها دوزهای50 و100 عصاره ارتشاح لنفوسیتی را نسبت به گروه جنتامایسین کاهش داد(05/0 >P). مصرف جنتامایسین سبب افزایش مقدار MDA سرم در مقایسه با گروه کنترل شد(05/0 >P). تنها دوز 100 عصاره توانست در مقایسه با سایر گروه های درمانی مقدار MDA سرمی را کاهش دهد(05/0 >P).مصرف هرسه دوز عصاره افزایش کراتی‌نین سرم ناشی از جنتامایسین را کاملا مهار نمود(001/0>P). تنها دوز50عصاره توانست افزایش اوره سرمی ناشی از مصرف جنتامایسین را کاهش دهد(05/0 >P).

Title: تعیین تأثیر تمرینات استقامتی و قدرتی بر میزان هموگلوبین گلکوزیله و قند خون ناشتای بیماران مبتلا به دیابت نوع دوم

http://yafte.lums.ac.ir/article-1-565-fa.html

مقدمه: پژوهش حاضر با هدف تعیین تأثیر تمرینات استقامتی و قدرتی بر میزان هموگلوبین گلکوزیله و قند خون ناشتای بیماران دیابتی نوع دوم در سال 88 در شهر خرم آباد انجام شده است. بحث و نتیجه‌گیری: این مطالعه نشان می دهد تمرین استقامتی باعث کاهش سطح هموگلوبین گلیکوزیله شده، درصورتیکه تمرینات قدرتی میزان آن را به طور معنی داری کاهش نداد و به نظر می رسد این تمرینات بیشتر باعث تغییر معنادار این فاکتور شده است. یافته‌ها: 29 نفر از آزمودنی ها در سه گروه تمرینات را تا پایان انجام دادند. میانگین سن، وزن، شاخص توده بدنی و میزان مصرف دارو در سه گروه تفاوت معنی دار آماری نشان نداد (76/0p=).کاهش هموگلوبین گلیکوزیله در گروه استقامتی قبل و پس از آزمون، تفاوت معنی داری نشان داد (02/0p= ). اما در گروه کنترل و تمرینات قدرتی، تغییرات تفاوت معنی دار نشان نداد . قند خون ناشتا پس آزمون در گروه استقامتی و قدرتی کاهش یافت و این تفاوت معنی دار بود، اما در گروه کنترل تفاوت معنی داری نشان نداد. مواد و روش‌ها: پژوهش حاضر مطالعه ای نیمه تجربی است که در بین 30 نفر از مردان مبتلا به دیابت نوع 2 مراجعه کننده به آزمایشگاههای شهر خرم آباد، پس از غربالگری و مصاحبه حضوری با روش نمونه گیری هدفمند انجام شده است.آزمودنی ها پس از تکمیل پرسشنامه سوابق پزشکی و رضایتنامه به طور تصادفی به سه گروه تمرین استقامتی، قدرتی و گروه کنترل تقسیم شدند.شرایط شرکت در پژوهش سن بالای 30 سال قند خون 150-250، فقدان هرگونه بیماری مزمن دیگر و سابقه ورزشی طی 3 ماه گذشته، سکته قلبی ، پرفشاری خون شدید و عوارض دیابت و...بودند. قبل از تمرینات از آزمودنی ها که به مدت 8-12 ساعت ناشتا بودند نمونه گیری خون انجام شد. تمرینات قدرتی و استقامتی به مدت 8 هفته و زیر نظر مربی مربوطه اجرا شدند.تمرینات قدرتی شامل 10 حرکت برای عضلات و برای هر حرکت سه تا چهار دور و هر دور، 10 تا 12تکرار در نظر گرفته شد و اصل اضافه بار در طول 8 هفته رعایت گردید. تمرینات استقامتی شامل دویدن بر روی دستگاه تردمیل با 60 % حداکثر ضربان قلب بود. 10نفردر گروه کنترل قرار داشتند که تا پایان تحقیق به توصیه ها عمل نمودند. پس از اتمام دوره هشت هفته ای تمرینات، پس آزمون (آزمایش خون) انجام شد . ارزیابی اطلاعات توسط نرم افزار آماری SPSS انجام پذیرفت. جهت مقایسه میانگین ها (پیش آزمون، پس آزمون) از تی جفت شده استفاده گردید.

Title: ارتباط برهمکنش‌های KIR-HLA با بیماری‌ها

http://yafte.lums.ac.ir/article-1-566-fa.html

پذیرنده های شبه ایمونوگلبولینی سلول کشنده (KIR) یک خانواده تازه کشف شده از پذیرنده های فعال کنندگی و مهاری است که عملکرد سلول کشنده طبیعی (NK) را کنترل می‌کند. KIR به عنوان یک خانواده متنوع از پذیرنده‌هایی است که به سرعت به وسیله دوپلیکاسیون ژنی و وقایع نوترکیبی تکامل یافته است. در ابتدا این یافته ها برای یک خانواده ژنی درگیر در پاسخ ایمنی ذاتی غیر منتظره بود. مولکول‌های آنتی‌ژن لکوسیت انسان (HLA) کلاس I به عنوان لیگاندهایی برای KIR عمل می‌کنند. برخی مطالعات ارتباط با بیماری نقش برهمکنش‌های این لوکوس‌ها را در بیماری‌های عفونی، اختلالات خودایمنی/التهابی، سرطان و تولیدمثل نشان داده است. نتایج عملکردی حاصله از مکانیسمی بر اساس یک طیف از مهار تا فعالیت از طریق ژنوتیپ‌های مختلف ترکیب KIR-HLA در بیماری‌ها حمایت می‌کند. این مقاله مروری خصوصیات اصلی این ژن‌ها را خلاصه کرده و بحث می‌کند که چگونه ممکن است آنها هم در پاتوژنز بیماری و هم در بهبود آن درگیر باشند.

Title: مهاجرت IUD به داخل مثانه وتشکیل سنگ بر روی آن

http://yafte.lums.ac.ir/article-1-567-fa.html

مهاجرت نابجای IUD به داخل مثانه وپیشابراه وتشکیل سنگ بر روی آن ازعوارض نادرIUD می‌باشد. در بررسی سیستوسکوپی و گرافی اولیه بر روی دو خانم 38 و45 ساله با سنگ راجعه مثانه به علت IUD که حدود 6-5 سال قبل گذاشته شده وبا علائم تحریکی ادراری مراجعه کرده بودند، اثری از IUD ونفوذ آن به داخل سنگ مشاهده نشد. سنگ ها بوسیله الکترو هیدرولیک شکسته شد. در مراجعات بعدی ودقت بیشتر مشخص شد که شاخ IUD حدود 5/0 سانتی متر به داخل مثانه نفوذ و موجب ایجاد سنگ شده بود،چون با سیستوسکوپ IUD قابل خارج شدن نبود جهت پیشگیری از عود بعدی وفیستول مثانه به واژن با سیستوستومی IUD سنگ ها بیرون آورده شد که درپیگیری2ساله هیچ عارضه ای مشاهده نگردید.
